# Regional Homogeneity in Patients With Mild Cognitive Impairment: A Resting-State Functional Magnetic Resonance Imaging Study

**DOI:** 10.3389/fnagi.2022.877281

**Published:** 2022-04-14

**Authors:** Yu-Qian Wu, Yi-Ning Wang, Li-Juan Zhang, Li-Qi Liu, Yi-Cong Pan, Ting Su, Xu-Lin Liao, Hui-Ye Shu, Min Kang, Ping Ying, San-Hua Xu, Yi Shao

**Affiliations:** ^1^Department of Ophthalmology and Neurology, The First Affiliated Hospital of Nanchang University, Nanchang, China; ^2^Department of Ophthalmology, Massachusetts Eye and Ear, Harvard Medical School, Boston, MA, United States; ^3^Department of Ophthalmology and Visual Sciences, The Chinese University of Hong Kong, Sha Tin, Hong Kong SAR, China

**Keywords:** rs-fMRI, mild cognitive impairment, ReHo, spontaneous brain activity, Alzheimer’s disease

## Abstract

**Objective:**

To analyze the potential changes in brain neural networks in resting state functional magnetic resonance imaging (rs-fMRI) scans by regional homogeneity (ReHo) in patients with mild cognitive impairment (MCI).

**Methods:**

We recruited and selected 24 volunteers, including 12 patients (6 men and 6 women) with MCI and 12 healthy controls matched by age, sex, and lifestyle. All subjects were examined with rs-fMRI to evaluate changes in neural network connectivity, and the data were analyzed by ReHo method. Correlation analysis was used to investigate the relationship between ReHo values and clinical features in different brain regions of MCI patients. The severity of MCI was determined by the Mini-Mental State Examination (MMSE) scale.

**Results:**

The signals of the right cerebellum areas 4 and 5, left superior temporal, right superior temporal, left fusiform, and left orbital middle frontal gyri in the patient group were significantly higher than those in the normal group (*P* < 0.01 by *t*-test of paired samples). The signal intensity of the right inferior temporal and left inferior temporal gyri was significantly lower than that of the normal group (*P* < 0.01). The ReHO value for the left inferior temporal gyrus correlated negatively with disease duration, and the value for the right inferior temporal gyrus correlated positively with MMSE scores.

**Conclusion:**

Mild cognitive impairment in patients with pre- Alzheimer’s disease may be related to the excitation and inhibition of neural networks in these regions. This may have a certain guiding significance for clinical diagnosis.

## Introduction

Cognitive decline is common in older adults, including dementia, delirium, depression, language problems, inattention, and low literacy levels (D’Atri et al., 2021). Mild cognitive impairment (MCI) is an intermediate stage of cognitive decline between normal aging and dementia in which people have memory problems or other cognitive abnormalities but have not yet reached the severity of dementia; therefore, the condition has little impact on daily living. MCI is common in the elderly, with a prevalence at age 65 of 6%. Because of the slow progression of the disease, a simple history and neurological examination alone are not enough to confirm the diagnosis. Studies have shown that 37–80% of dementia is not clinically diagnosed, suggesting that cognitive impairment is difficult to detect without screening tools (D’Atri et al., 2021). The most common cause of mild cognitive impairment is Alzheimer’s disease (AD) (Yukimasa, 2017).

AD is a series of primary degenerative encephalopathies occurring in middle-aged and older adults (Yukimasa, 2017). The incidence of AD is closely related to several factors such as age, genetics, and environment that produce common pathological changes concerning metabolism, blood vessels, and inflammation (Yukimasa, 2010). In terms of metabolism, previous studies that explored the independent and reciprocal effects of curcumin on the brain and liver have shown that curcumin injection prior to Aβ deposition can prevent AD in APP/PS1 mice, suggesting that curcumin may significantly affect the elimination of Aβ42 in cerebral blood transport and peripheral circulation (Yepes, 2021). Cerebrovascular disease mainly presents symptoms of progressive memory loss, MCI, distraction, affective disorder, personality changes, and other characteristics. Alzheimer’s disease often presents as a persistent disorder of high-level neural function (Wu et al., 2021). It has been reported that in APP/PS1 mice, a double transgenic mouse model of AD, female mice developed Aβ plaque load in the dentate gyrus layer at an early stage. There was also a significant neuroinflammatory activation of astrocytes and microglia (Jung et al., 2019). In addition, some studies have shown that magnetic resonance imaging (MRI)-based assessment of brain atrophy can be used to evaluate and stage Alzheimer’s disease, which laid a foundation for the specific neuroimaging changes in the brain of our study (Bijttebier et al., 2021). Researchers have indicated that it is now possible to measure tau and amyloid beta (Aβ) protein in the brain. Analyses of the association among neuroimaging findings, clinical phenotype, and age were performed as a way to investigate how different neuroimaging modalities relate to disease mechanisms; hence, it is possible to elaborate on the specific cause of, as well as the course of, Alzheimer’s disease (Benitez et al., 2021; Roberts et al., 2021).

**FIGURE 1:**
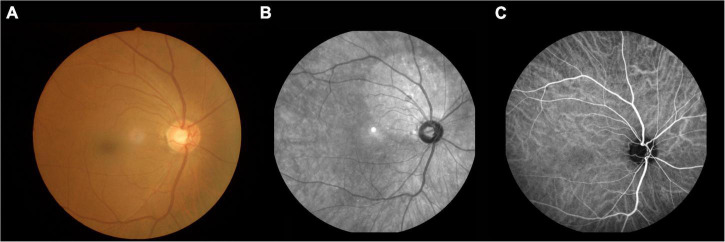
An example of MCI. **(A)** Fundus photography; **(B)** fundus fluorescein angiography; **(C)** indocyanine green angiography. MCI, mild cognitive impairment.

Aβ has been shown to accumulate in the retina of patients with MCI, and this phenomenon may appear before accumulation in the brain (Mei et al., 2020). Detecting an accumulation of Aβ in the eye may prove to be a useful clinical method for early diagnosis of AD before the onset of clinical symptoms, but the relevant diagnostic approach needs to be further developed (Figure 1).

Despite these advances, there is still a lack of strong evidence that can be used as a basis for the clinical diagnosis of AD. MRI is a non-invasive diagnostic technique used in the medical and biomedical fields to evaluate nervous system structure and neurological function (Zang et al., 2004). Resting-state functional magnetic resonance imaging (rs-fMRI) allows measurements of brain activity at rest, and its use has evolved rapidly in recent years (Jiang et al., 2021). Regional homogeneity (ReHo) is a technique for analyzing rs-fMRI results that has been normally applied in clinical practice. Recent studies on the application of the ReHo method to analyze neuroimaging changes are shown in Table 1 (Tononi et al., 1998; Xiang et al., 2018; Liao et al., 2019; Shao et al., 2019; Wen et al., 2019; Li et al., 2020; Guo et al., 2021). By calculating the Kendall coefficient consistency of voxel dynamic fluctuation time-series in a specific cluster, the local synchronization of spontaneous rs-fMRI signals is explored, which represents essential data for normal brain activity (Fang et al., 2021; Gaubert et al., 2021). Brain dysfunction in patients may lead to changes in synchronization of neurons in the brain, which adversely affects neural information processing, and thus reflects numerical differences from people with normal brain activity.

**TABLE 1:** REHO method applied in neurogenic disease and ophthalmologic.

References	Years	Disease
Guo et al. (2021)	2019	Classical trigeminal neuralgia
Xiang et al. (2018)	2019	Retinal vein occlusion
Wen et al. (2019)	2019	Diabetic retinopathy
Li et al. (2020)	2019	Strabismus and amblyopia
Liao et al. (2019)	2021	Diabetic optic neuropathy
Shao et al. (2019)	2021	Thyroid-associated ophthalmopathy
Tononi et al. (1998)	2020	Parkinson

The severity of dementia can be determined based on the results of neuropsychological assessments. The commonly used clinical tool is the Mini-Mental State Examination (MMSE) Scale (0–30 points; lower scores represent more severe cognitive impairment) (Cheng et al., 2019).

Receiver operating characteristics (ROC) curve analysis is a tool that can describe diagnostic accuracy in medical research, and the area under the curve (AUC) often serves as one of the criteria for comparison. A larger AUC implies a higher correlation, which in turn represents a higher diagnostic accuracy (Dong et al., 2021).

## Materials and Methods

### Participants

A total of 24 volunteers were chosen for this study, including 12 patients (6 men and 6 women) with MCI and 12 healthy controls (HC) matched by age, gender, and lifestyle from the Ophthalmology Department of the First Affiliated Hospital of Nanchang University Hospital. The inclusion criteria for participants with MCI were as follows: (1) Age ≥ 45 years; (2) chief complaint of memory decline and MMSE < 27 points; (3) Barthel index for ability to perform activities of daily living ≥ 90 points; and (4) recent cranial MRI suggesting no parenchymal brain lesions. The exclusion criteria for participants with MCI were the following: (1) vascular dementia, Parkinson dementia, frontotemporal lobar degeneration and other types of dementia, cognitive impairment due to other causes, acute cerebral hemorrhage, cerebral infarction, and intracranial space occupying lesions; (2) other psychiatric disorders, such as severe affective disorder or current evidence of depression; (3) visual and hearing impairment; and (4) severe dementia.

Inclusion criteria for the HC group were as follows: (1) Age ≥ 45 years; (2) routine brain MRI without obvious abnormalities; (3) no memory problems and an MMSE score ≥ 27 points; (4) no neurological, psychiatric, and cardiovascular diseases; (5) no drug or alcohol addiction; and (6) able to undergo MRI.

The medical ethics committee of the First Affiliated Hospital of Nanchang University approved all research methods which were in accordance with the 1964 Declaration of Helsinki and its later amendments or comparable ethical standards. The purpose, method, and potential risks of participating in the study were explained to all participants, and all participants signed an informed consent form.

### Magnetic Resonance Imaging Parameters

All subjects were scanned with a 3-Tesla magnetic resonance scanner (Trio, Siemens, Munich, Germany). They were instructed to keep their eyes closed, but to remain awake and relaxed until the end of the scan. Using a three-dimensional spoiled gradient-recalled echo sequence in the MRI, relevant data was then obtained. Imaging parameters of the T1 and T2 sequences for 176 traverse images were as follows: TR = 1,900 ms, TE = 2.26 ms, thickness = 1.0 mm, gap = 0.5 mm, acquisition matrix = 256 × 56, field of view = 250 × 250 mm, and flip angle = 9°. Imaging parameters for 240 functional images were as follows: TR = 2,000 ms, TE = 30 ms, thickness = 4.0 mm, gap = 1.2 mm, acquisition matrix = 64 × 64, flip angle = 90°, field of view = 220 × 220 mm, and 29 axial. Scanning times were 5 and 10 min, respectively.

### Data Analysis for Resting State Functional Magnetic Resonance Imaging

The MRIcro software^1^ was used to organize the data, including classifying the data and deleting the incomplete data. Moreover, Statistical Parametric Mapping (SPM; The MathWorks, Inc.)^2^ and the Data Processing Assistant for rs-fMRI software (DPARSFA; version 4.0; Institute of Psychology, Chinese Academy of Sciences)^3^ were used to analyze the data. The regions of interest (ROI) in patients with MCI and HC were divided with the RESTing-state fMRI data analysis toolkit (REST). The echo planar imaging was used to standardize the rs-fMRI images, which met the spatial standards of the Montreal Institute of Neurology (MNI).

Participant 3 was excluded because his head movements were > 3 mm and the rest of his head movements were < 3 mm and well matched.

### Statistical Analysis

We used SPSS software, version 22.0 (International Business Machines Corporation, Inc. (IBM), Armonk, New York, United States) in this study to analyze changes in brain neural signal fluctuations (i.e., ReHo values). Paired samples *t*-tests were used to test whether the two means were the same overall. In general, the larger the *t*-value, the more likely it is to be statistically relevant. We considered results significant if *P*<0.05. Multiple comparison correction used Gaussian Random Field (GRF) with a voxel level threshold of 0.005 and a cluster level threshold of 0.05 for two-sided tests. ROC curve analysis was used to compare the rs-fMRI values of the two groups. This represents how accurate rs-fMRI values are in the diagnosis of MCI. The AUC was of primary interest, and represents the diagnostic yield in this analysis. AUC > 0.9 was considered as a numerical value that represents high diagnostic accuracy.

### Brain-Behavior Correlation Analysis

We collected clinical data from all study participants, including MMSE scores and disease duration, to find correlations between these data and the mean ReHo values of the different brain regions studied. Pearson correlation was analyzed with GraphPad Prism 8 software (GraphPad Inc., San Diego, CA, United States) to evaluate and graph the linear correlation between MMSE scores, duration of MCI and ReHo values.

## Results

### Demographics

Subject 3’s data have been deleted due to excessive head movement. There was no significant difference in mean age between the MCI and HC groups (64.27 ± 7.35 and 64.00 ± 6.18 yr, respectively; *P* = 0.924). There was no significant difference in the male to female ratio between the MCI and HC groups. The average MMSE scores of the MCI group was 22.09 ± 4.11 (*P* < 0.001). The average duration of the MCI was 13.55 ± 9.59 months (*P* < 0.001). The average S100β of MCI was 2.76 ± 1.09 (*P* < 0.001). A detailed summary of the data is presented in Table 2.

**TABLE 2:** Demographic characteristic of the enrolled subjects.

Condition	MCI	HC	*t*	*P*
Subject	11	12	NA	NA
Age (y)	64.27 ± 7.35	64.00 ± 6.18	0.097	0.924
Gender (M:F)	5:6	6:6	NA	NA
Duration (month)	13.55 ± 9.59	0	4.687	<0.001
SBP	128.45 ± 12.47	130.5 ± 10.83	–0.421	0.678
DBP	75.27 ± 12.39	76.17 ± 10.57	–0.187	0.854
HR	69.27 ± 9.48	70.42 ± 8.36	–0.308	0.761
Barthel index	99.55 ± 1.51	100	–1.000	0.341
Best-corrected V A-left eye	0.30 ± 0.10	0.23 ± 0.06	2.181	0.041
Best-corrected V A-right eye	0.26 ± 0.12	0.21 ± 0.08	1.311	0.204
S100β	2.76 ± 1.09	0.18 ± 0.09	–8.211	<0.001
MMSE	22.09 ± 4.11	27.83 ± 2.52	–4.081	<0.001

*MCI, mild cognitive impairment; HC, Healthy control; NA, not applicable; SBP, systolic blood pressure; DBP, diastolic blood pressure; HR, heart rate.*

### Regional Homogeneity Differences

**FIGURE 2:**
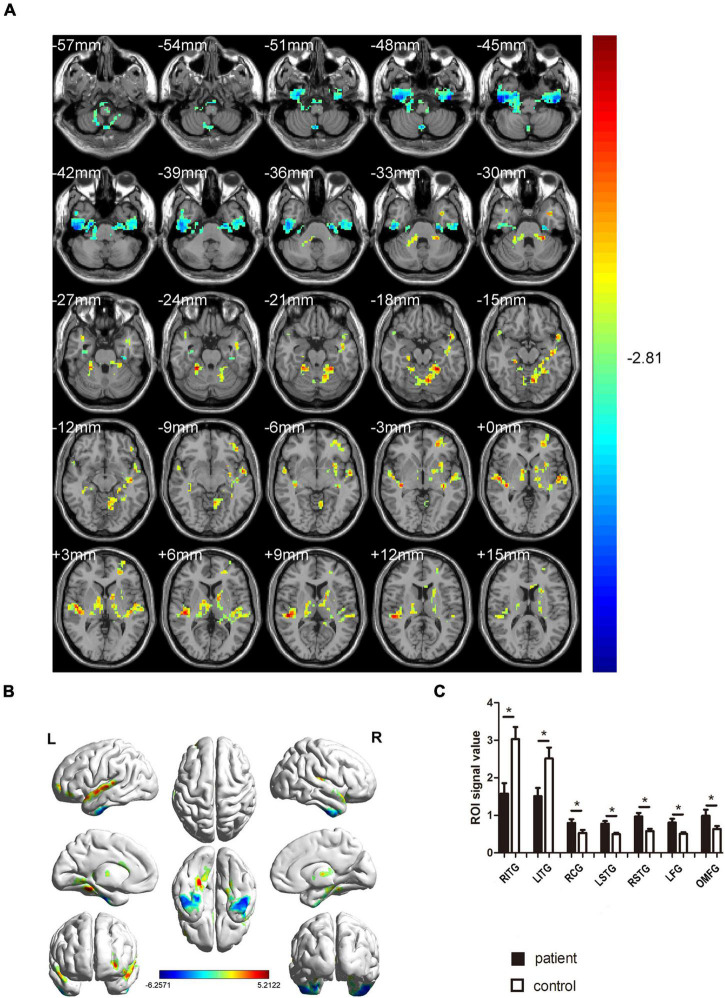
Comparison of ReHo values in MCI and HC groups. **(A)** Differences in ReHo were found in RCG, LSTG, RSTG, LFG, and OMFG, RITG, and LITG. **(B)** The stereoscopic form of the cerebrum. The red area indicates an increase in ReHo value; the blue indicates a decrease in ReHo value. (GRF correction, the cluster-level: *P* < 0.05; two-tailed, with voxel level *P* < 0.005). **(C)** The Mean ReHo value between MCIs and control group. ReHo, regional homogeneity; MCI, mild cognitive impairment; HC, healthy controls. *P < 0.05 Independent t-tests comparing two groups.

In contrast with the HC group, the ReHo values of MCI patients for the right inferior temporal gyrus (RITG) and the left inferior gyrus (LITG) exhibited significantly lower in Figures 2A,B (blue) and Table 3. At the same time, the ReHo values of MCI patients were increased in the right cerebellar gyrus areas 4 and 5 (RCG), left superior temporal gyrus (LSTG), right superior temporal gyrus (RSTG), left fusiform gyrus (LFG), and left orbital middle frontal gyrus (OMFG) as shown in Figures 2A,B (red) and Table 3 (*P* < 0.01, using GRF theory for multiple comparisons, *z* > 2.3, *P* < 0.01, cluster > 40 voxels, AlphaSim has been corrected).

**TABLE 3:** Brain areas with different ReHo values between MCIs and HCs.

Brain areas(aal)	MNI coordinates			BA	Number of voxels	*T*-value
	X	Y	Z			
HC > MCI						
RITG	48	–15	–45	–	114	–6.2571
LITG	–45	–15	–48	–	65	–5.4858
HC < MCI						
RCG	24	–39	–24	98	117	5.2122
LSTG	–54	–9	0	81	136	4.7359
RSTG	51	–27	9	82	125	4.9241
LFG	–24	–42	–18	55	312	4.8765
OMFG	–27	48	–3	–	121	4.2007

*The statistical threshold was set at the voxel level with P < 0.05 for multiple comparisons using Gaussian Random Field theory (z > 2.3, P < 0.01, cluster > 40 voxels, AlphaSim corrected).*

*ReHo, regional homogeneity; BA, Brodmann area; HCs, healthy controls; MNI, Montreal Neurological Institute. RITG, the right inferior temporal gyrus; LITG, the right inferior temporal gyrus; RCG, the cerebellum superior; LSTG, the left superior temporal gyrus; RSTG, the right superior temporal gyrus; LFG, the left fusiform gyrus; OMFG, the left orbital middle frontal gyrus.*

### Receiver Operating Characteristics Curve

**FIGURE 3:**
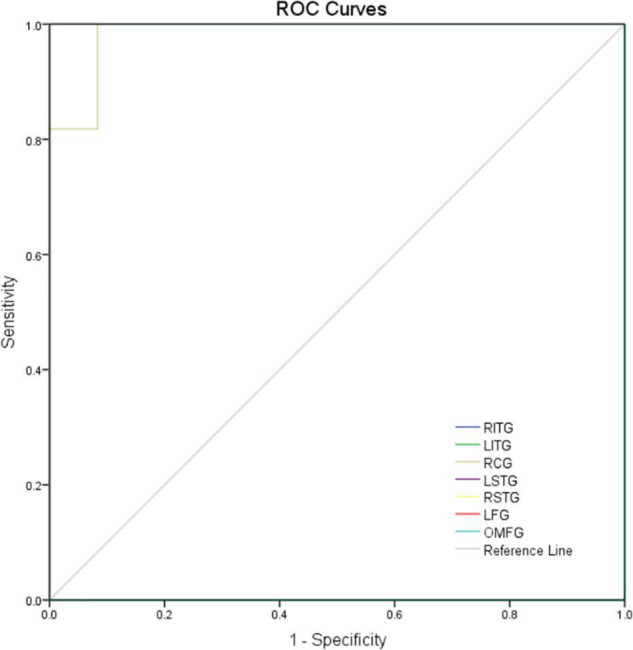
ROC curve analysis of the mean ReHo values for altered brain regions. The area under the ROC curve were 1.000, (*p*<0.0001; 95% CI: 1.000–1.000) for LITG, RITG, LSTG, RSTG, LFG, and OMFG; 0.985 (*p* < 0.01; 95% CI: 0.946–1.000) for RCG. AUC, area under the curve; ROC, receiver operating characteristic.

Considering the abnormal activity of certain brain regions in MCI patients, we analyzed the diagnostic value of ReHo for MCI by ROC curve analysis. In this study, the AUC value was 1.000 (*P*<0.0001; 95% CI: 1.000–1.000) for LITG, RITG, LSTG, RSTG, LFG, and OMFG; meanwhile the AUC value was 0.985 (*P* < 0.01; 95% CI: 0.946–1.000) for RCG (Figure 3). These results show that ReHo values in these brain regions showed significant differences between patients with MCI and healthy controls.

### Correlation Analysis

**FIGURE 4:**
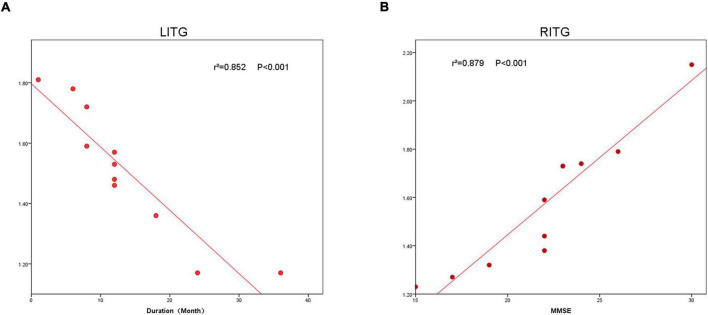
The correlation between the ReHo value of LITG, RITG and the duration **(A),** and MMSE **(B)**. In the Alzheimer’s disease group, the ReHo value of LITG showed a negative correlation with duration (*r*^2^ = 0.852, *P* < 0.0001). The ReHo value of the RITG was positively correlated with MMSE (*r*^2^ = 0.879, *P* < 0.0001). ReHo, regional homogeneity; MCI, mild cognitive impairment.

In patients with MCI, the ReHO value in LITG (*r*^2^ = 0.852, *P* < 0.001) correlated negatively with disease duration, and the ReHO value in RITG (*r*^2^ = 0.879, *P* < 0.001) correlated positively with the MMSE (Figure 4).

## Discussion

The rs-fMRI provides insight into abnormal electrical activity in patients’ brains during a disease state. In this study we use the ReHo method to measure abnormal activity in specific brain regions, and has been widely used in the diagnosis, treatment, and prognosis of various craniocerebral injuries, ophthalmic diseases, and related neurological diseases (Gu et al., 2022).

Hypointensity in the LITG area may be associated with auditory naming disorder in mild cognitive impairment; however, there are few specific functional studies on LITG (Takamura et al., 2021). Some studies found that activation of language networks and task-related functional connectivity exist in the left temporal lobe, and there was activation of the LITG area during auditory naming in the clinical naming experiment (Mitchell et al., 2020). In some studies, rs-fMRI values of individuals with cognitive decline due to sleep deprivation are represented in LITG, specifically by attenuated alterations in the effective connectivity of LITG with other brain regions (Lyu et al., 2021). Previous studies have shown that significant genetic overlap exists between hearing loss and AD, and that a polygenic risk score for AD can significantly predict hearing loss in an independent cohort, suggesting that there is a correlation between damage in this brain region and genetic risk, but the specific correlation remains to be further studied (Kang et al., 2021). In our study, we demonstrated that the MCI patients show decreased ReHo values in the LITG, which indicates auditory naming dysfunction. In addition, we found a negative correlation between the ReHo signal value of LITG and the duration of MCI; thus, the longer the duration of the disease, the lower the ReHo signal value of this brain region.

**FIGURE 5:**
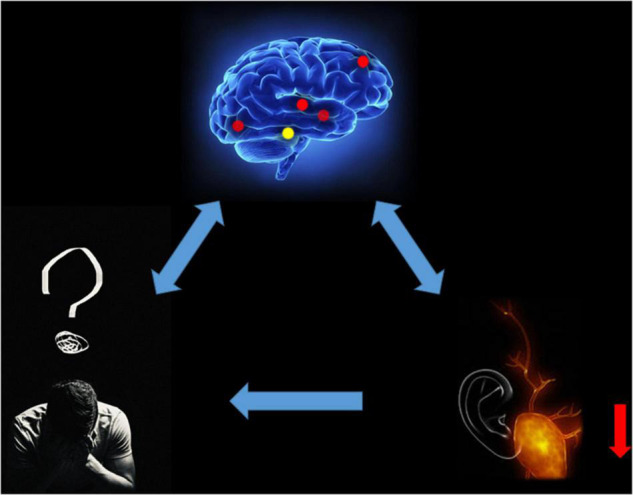
Relationship between MRI images and clinical manifestations in MCI. MCI, mild cognitive impairment.

Some studies have shown that the RITG may be related to the consciousness of important emotional significance. Alfredson et al. (2010) studied the changes in temporal lobe blood flow in volunteers who listened to standard music vs. important emotional music. Measurements were made during silence, individually selected emotional music, and standard neutral music. The RITG showed a significant (*p* < 0.01) increase in regional cerebral blood flow (rCBF) when the emotional music was compared to silence. A temporal lobe asymmetry (right > left) during emotional music was also significant (*p* < 0.01) (Alfredson et al., 2010). In addition, another study demonstrated increased signal in the RITG and a positive correlation with alertness in patients with right temporal lobe epilepsy. The significant decrease in RITG signal found in this study for the MCI group may be related to the reduced response to important affective meanings seen in patients with MCI (Gellersen et al., 2021). This may be related to the abnormal mental behavior of AD patients; that is, positive mental symptoms such as agitation, anxiety, and mania as well as negative mental symptoms such as depression and indifference caused by cerebral cortex damage (Figure 5). Furthermore, the ReHo value of RITG showed a significant positive correlation with the MMSE score of MCI patients, and data processing also showed a significant negative correlation between the signal intensity of this brain region and the length of the disease. This result implies that the ReHo value of RITG can be indirectly correlated with MMSE scores to determine the severity of MCI.

**FIGURE 6:**
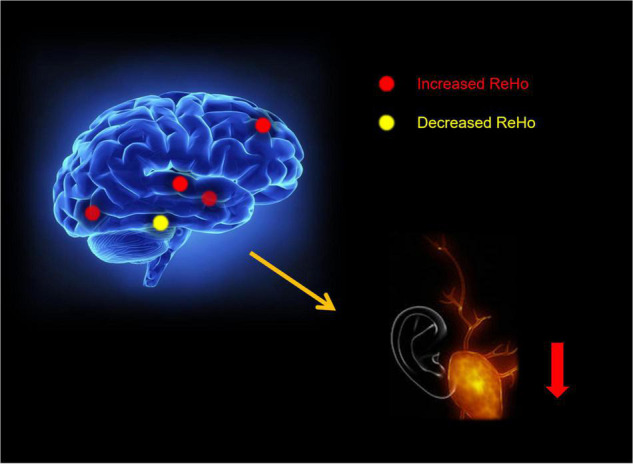
The hearing loss results of brain activity.

We speculate that ReHo values in patients with MCI decrease in RITG and LITG represented by the auditory naming and hearing loss, and important affective disorders may be associated with abnormal mental activity. Emotions such as agitation, mania or depression, apathy and hearing loss caused by decreased quality of life may also affect the degree of MCI (Figure 6).

The cerebellum in general has control over sensorimotor and vestibular components, and additionally influences cognitive, emotional, and autonomic function (Xu et al., 2021). A previous meta-analysis of cerebellar gray matter loss in normal aging and AD by Shah et al. (2013) found that gradient 3 (which captures lateralization differences in cognitive function) was significantly different in normal aging compared with AD, indicating slight functional differences between left and right cerebellar atrophy regions. The exact function of the RCG region is not fully understood; however, compared with the HCs, the ReHo value of this region was significantly increased in this study, which may be related to the changes in autonomic and cognitive function of MCI patients.

A major function of the superior temporal gyrus is extracting meaningful linguistic features from speech inputs, and is strongly modulated by learning knowledge and perceived goals (Zhongwei et al., 2017). There is some evidence that the right STG functions in allocentric neglect deficits (Mao et al., 2021). The significantly higher signal in this region in the MCI group in the present study may be related to the altered perception of language in patients with MCI.

The fusiform gyrus may be associated with face processing, and the high signal expression in LFG found in this study may be associated with altered face processing ability in patients with MCI (Zuo et al., 2013). Gerłowska et al. (2021) stated that further research on facial expression patterns of the older adults can reduce misunderstandings and improve patients’ quality of life. Therefore, we can speculate that stimulation targeting of these brain regions can significantly improve the facial expression patterns of AD patients and thus improve their quality of life.

There are few studies on the function of OMFG. Some studies suggest that the signal changes of OMFG are related to the changes in neurological function of patients with anxiety and depression. Previous studies have found that patients with severe anxiety and depression had significantly lower amplitude of low-frequency fluctuation values in the brain regions, means that the brain regions activity obviously changed (Zhao et al., 2019). In our study, the MCI patients ReHo values for the OMFG were significantly higher compared to HCs: thus, we hypothesize that it is possible to identify patients with complications of anxiety and depression by monitoring the changes in signal values in this brain region. However, we did not evaluate anxiety and depression scores for study participants that would allow us to prove a relationship between the degree of signal changes and the degree of anxiety and depression; thus, this association remains to be studied further. Please refer to Table 4 for the changes of ReHo values in brain regions and their effects on brain function.

**TABLE 4:** Brain areas alteration and its potential impact.

Brain areas	Experimental result	Function
Inferior temporal gyrus	HCs > MCIs	Related to cognitive learning and object memory, emotional processing
Fusiform gyri	HCs < MCIs	Face recognition, Secondary classification and recognition of objects
Superior temporal gyri	HCs < MCIs	Responsible for extracting meaningful linguistic features from speech inputs
Right cerebellum areas 4 and 5	HCs < MCIs	control over sensorimotor and vestibular components
Left orbital middle frontal gyri	HCs < MCIs	Related to the changes of neurological function in patients with anxiety and depression

*HCs, healthy controls; MCI, mild cognitive impairment.*

This study has some limitations. First of all, the different lengths of scanning time and the occurrence of multiple body movements during the rs-fMRI scanning process will produce different qualities of images in the scanning results; thus, there are avoidable errors in the obtained values. Reducing these individual differences may improve the specificity and accuracy of our analysis. Secondly, the sample size was small, and further and more accurate studies with a larger sample size are needed to validate our findings. This may explain why we failed to find positive results in the process of verifying the correlation between the course of disease and MMSE score and the degree of signal value change for each brain region.

Our results showed that all MCI patients had varying degrees of increased or decreased abnormal electrical signals in rs-fMRI imaging of the brain regions we explored, which may reflect the pathogenesis of AD and identify potential risk factors. These ReHo values can be used to help clinicians diagnose and assess the severity of MCI.

## Conclusion

This paper analyzed the alterations in ReHo of fMRI signals in a resting state. Alterations in the functional connectivity patterns of regions of interest and whole brain analyses were in turn analyzed to deduce the pathogenesis of MCI as well as disease progression. This may provide some diagnostic basis for clinical practice, as well as some significance for patient prognosis.

## Data Availability Statement

The raw data supporting the conclusions of this article will be made available by the authors, without undue reservation.

## Ethics Statement

The studies involving human participants were reviewed and approved by the First Affiliated Hospital of Nanchang University. The patients/participants provided their written informed consent to participate in this study.

## Author Contributions

Y-QW, Y-NW, and L-JZ analyzed the data and draft the manuscript. L-QL, Y-CP, TS, and X-LL assisted with data interpretation and figure composing. H-YS, MK, PY, and S-HX collected the data. YS conceived, designed and directed the study, and final revised and approved the manuscript. All authors contributed to the article and approved the submitted version.

## Conflict of Interest

The authors declare that the research was conducted in the absence of any commercial or financial relationships that could be construed as a potential conflict of interest.

## Publisher’s Note

All claims expressed in this article are solely those of the authors and do not necessarily represent those of their affiliated organizations, or those of the publisher, the editors and the reviewers. Any product that may be evaluated in this article, or claim that may be made by its manufacturer, is not guaranteed or endorsed by the publisher.

## Footnotes

1
www.MRIcro.com

2
http://www.fil.ion.ucl.ac.uk/spm

3
http://rfmri.org/DPARSF

## References

[B1] Alfredson B. B. Risberg J. Hagberg B. Gustafson L. (2010). Right temporal lobe activation when listening to emotionally significant music. *Appl. Neuropsychol.* 11 161–166. 10.1207/s15324826an1103_4 15590350

[B2] Benitez D. P. Jiang S. Wood J. Wang R. Hall C. M. Peerboom C. (2021). Knock-in models related to Alzheimer’s disease: synaptic transmission, plaques and the role of microglia. *Mol. Neurodegener.* 16:47. 10.1186/s13024-021-00457-0 34266459 PMC8281661

[B3] Bijttebier S. Theunis C. Jahouh F. Martins D. R. Verhemeldonck M. Grauwen K. (2021). Development of immunoprecipitation – two-dimensional liquid chromatography – mass spectrometry methodology as biomarker read-out to quantify phosphorylated tau in cerebrospinal fluid from Alzheimer disease patients. *J. Chromatogr.* 1651:462299. 10.1016/j.chroma.2021.462299 34107398

[B4] Cheng J. Yang H. Zhang J. (2019). Donepezil’s effects on brain functions of patients with Alzheimer disease: a regional homogeneity study based on resting-state functional magnetic resonance imaging. *Clin. Neuropharmacol.* 42 42–48. 10.1097/WNF.0000000000000324 30875345 PMC6426347

[B5] D’Atri A. Scarpelli S. Gorgoni M. Truglia I. Lauri G. Cordone S. (2021). EEG alterations during wake and sleep in mild cognitive impairment and Alzheimer’s disease. *iScience* 24:102386. 10.1016/j.isci.2021.102386 33981973 PMC8086022

[B6] Dong W. J. Su T. Li C. Q. Shu Y. Q. Shi W. Q. Min Y. L. (2021). Altered brain network centrality in patients with retinal vein occlusion: a resting-state fMRI study. *Int. J. Ophthalmol.* 14 1741–1747. 10.18240/ijo.2021.11.14 34804865 PMC8569561

[B7] Fang M. Strand K. Zhang J. Totillo M. Signorile J. F. Galvin J. E. (2021). Retinal vessel density correlates with cognitive function in older adults. *Exp. Gerontol.* 152:111433. 10.1016/j.exger.2021.111433 34091000 PMC8521640

[B8] Gaubert S. Houot M. Raimondo F. Ansart M. Corsi M. C. Naccache L. (2021). Machine learning approach to screen for preclinical Alzheimer’s disease. *Neurobiol. Aging* 105 205–216. 10.1016/j.neurobiolaging.2021.04.024 34102381

[B9] Gellersen H. M. Guell X. Sami S. (2021). Differential vulnerability of the cerebellum in healthy ageing and Alzheimer’s disease. *Neuroimage Clin.* 30:102605. 10.1016/j.nicl.2021.102605 33735787 PMC7974323

[B10] Gerłowska J. Dmitruk K. Rejdak K. (2021). Facial emotion mimicry in older adults with and without cognitive impairments due to Alzheimer’s disease. *AIMS Neurosci.* 8 226–238. 10.3934/Neuroscience.2021012 33709026 PMC7940111

[B11] Gu X. Q. Liu Y. Gu J. B. Li L. F. Fu L. L. Han X. M. (2022). Correlations between hippocampal functional connectivity, structural changes, and clinical data in patients with relapsing-remitting multiple sclerosis: a case-control study using multimodal magnetic resonance imaging. *Neural Regen. Res.* 17 1115–1124. 10.4103/1673-5374.324855 34558540 PMC8552851

[B12] Guo G. Y. Zhang L. J. Li B. Liang R. B. Ge Q. M. Shu H. Y. (2021). Altered spontaneous brain activity in patients with diabetic optic neuropathy: a resting-state functional magnetic resonance imaging study using regional homogeneity. *World J. Diabetes* 12 278–291. 10.4239/wjd.v12.i3.278 33758647 PMC7958477

[B13] Jiang Y. P. Yang Y. C. Tang L. Y. Ge Q. M. Shi W. Q. Su T. (2021). Altered spontaneous brain activity patterns in dysthyroid optic neuropathy: a resting-state fMRI study. *J. Integr. Neurosci.* 20 375–383. 10.31083/j.jin2002037 34258936

[B14] Jung F. Kazemifar S. Bartha R. Rajakumar N. (2019). Semiautomated assessment of the anterior cingulate cortex in Alzheimer’s disease. *J. Neuroimaging* 29 376–382. 10.1111/jon.12598 30640412

[B15] Kang D. W. Wang S. M. Um Y. H. Na H. R. Kim N. Y. Lee C. U. (2021). Distinctive association of the functional connectivity of the posterior cingulate cortex on memory performances in early and late amnestic mild cognitive impairment patients. *Front. Aging Neurosci.* 13:696735. 10.3389/fnagi.2021.696735 34276347 PMC8281268

[B16] Li M. G. Liu T. F. Zhang T. H. Chen Z. Y. Nie B. B. Lou X. (2020). Alterations of regional homogeneity in Parkinson’s disease with mild cognitive impairment: a prelimin ary resting-state fMRI study. *Neuroradiology* 62 327–334. 10.1007/s00234-019-02333-7 31822931

[B17] Liao X. L. Yuan Q. Shi W. Q. Li B. Su T. Lin Q. (2019). Altered brain activity in patients with diabetic retinopathy using regional homogeneity: a resting-state FMRI Study. *Endocr. Pract.* 25 320–327. 10.4158/EP-2018-0517 30995427

[B18] Lyu H. Jiao J. Feng G. Wang X. Sun B. Zhao Z. (2021). Abnormal causal connectivity of left superior temporal gyrus in drug-naïve first- episode adolescent-onset schizophrenia: a resting-state fMRI study. *Psychiatry Res. Neuroimaging* 315:111330. 10.1016/j.pscychresns.2021.111330 34280873

[B19] Mao Y. Liao Z. Liu X. Li T. Hu J. Le D. (2021). Disrupted balance of long and short-range functional connectivity density in Alzheimer’s disease (AD) and mild cognitive impairment (MCI) patients: a resting-state fMRI study. *Ann. Transl. Med.* 9:65. 10.21037/atm-20-7019 33553358 PMC7859805

[B20] Mei X. Yang M. Zhu L. Zhou Q. Li X. Chen Z. (2020). Retinal levels of amyloid beta correlate with cerebral levels of amyloid beta in young APPswe/PS1dE9 transgenic mice before onset of Alzheimer’s disease. *Behav. Neurol.* 2020:1574816. 10.1155/2020/1574816 33029254 PMC7532376

[B21] Mitchell B. L. Thorp J. G. Evans D. M. Nyholt D. R. Martin N. G. Lupton M. K. (2020). Exploring the genetic relationship between hearing impairment and Alzheimer’s disease. *Alzheimers Dement.* 12:e12108. 10.1002/dad2.12108 33005726 PMC7517507

[B22] Roberts S. Gardner C. Jiang Z. Abedi A. Buser Z. Wang J. C. (2021). Analysis of trends in lumbar disc degeneration using kinematic MRI. *Clin. Imaging* 79 136–141. 10.1016/j.clinimag.2021.04.028 33940491

[B23] Shah P. P. Spaldo N. Barrett A. M. Chen P. (2013). Assessment and functional impact of allocentric neglect: a reminder from a case study. *Clin. Neuropsychol.* 27 840–863. 10.1080/13854046.2013.783120 23560431 PMC3759518

[B24] Shao Y. Li Q. H. Li B. Lin Q. Su T. Shi W. Q. (2019). Altered brain activity in patients with strabismus and amblyopia detected by analysis of regional homogeneity: a resting state functional magnetic resonance imaging study. *Mol. Med. Rep.* 19 4832–4840. 10.3892/mmr.2019.10147 31059016 PMC6522834

[B25] Takamura Y. Fujii S. Ohmatsu S. Ikuno K. Tanaka K. Manji A. (2021). Interaction between spatial neglect and attention deficit in patients with right hemisphere damage. *Cortex* 141 331–346. 10.1016/j.cortex.2021.03.036 34126288

[B26] Tononi G. McIntosh A. R. Russell D. P. Edelman G. M. (1998). Functional clustering: identifying strongly interactive brain regions in neuroimaging data. *Neuroimage* 7 133–149. 10.1006/nimg.1997.0313 9558645

[B27] Wen S. M. Min Y. L. Yuan Q. Li B. Lin Q. Zhu P. W. (2019). Altered spontaneous brain activity in retinal vein occlusion as determined by regional homogeneity: a resting-state fMRI study. *Acta Radiol.* 60 1695–1702. 10.1177/0284185119845089 31023069

[B28] Wu X. Shen Q. Zhang Z. Zhang D. Gu Y. Xing D. (2021). Photoactivation of TGFβ/SMAD signaling pathway ameliorates adult hippocampal neurogenesis in Alzheimer’s disease model. *Stem Cell Res. Ther.* 12:345. 10.1186/s13287-021-02399-2 34116709 PMC8196501

[B29] Xu J. Dong H. Li N. Wang Z. Guo F. Wei J. (2021). Weighted RSA: an improved framework on the perception of audio-visual affective speech in left insula and superior temporal gyrus. *Neuroscience* 469 46–58. 10.1016/j.neuroscience.2021.06.002 34119576

[B30] Xiang C. Q. Liu W. F. Xu Q. H. Su T. Yong-Qiang S. Min Y. L. (2018). Altered spontaneous brain activity in patients with classical trigeminal neuralgia using regional homogeneity: a resting-state functional MRI study. *Pain Pract.* 19 397–406. 10.1111/papr.12753 30536573

[B31] Yepes M. (2021). The plasminogen activating system in the pathogenesis of Alzheimer’s disease. *Neural Regen. Res.* 16 1973–1977. 10.4103/1673-5374.308076 33642369 PMC8343336

[B32] Yukimasa T. (2010). “10P-E-6 the relationship between fractal dimension of brain CT images and the scores on the MMSE in senile dementia of Alzheimer type preliminary report(room E international session),” in *Proceedings of the Annual Conference of Biomedical Fuzzy Systems Association.* 10.24466/pacbfsa.23.0_341

[B33] Yukimasa T. (2017). Fractal analysis of brain CT image in Senile dementia of Alzheimer type (new development of SOFT science in BMFSA2009-AMAMI). *Int. J. Biomed. Soft Comput. Hum. Sci.* 16 81–86.

[B34] Zang Y. Jiang T. Lu Y. He Y. Tian L. (2004). Regional homogeneity approach to fMRI data analysis. *Neuroimage* 22 394–400. 10.1016/j.neuroimage.2003.12.030 15110032

[B35] Zhao P. Yan R. Wang X. Geng J. Chattun M. R. Wang Q. (2019). Reduced resting state neural activity in the right orbital part of middle frontal gyrus in anxious depression. *Front. Psychiatry* 10:994. 10.3389/fpsyt.2019.00994 32038329 PMC6987425

[B36] Zhongwei G. Xiaozheng L. Jiapeng L. Fuquan W. Hongtao H. Xingli C. (2017). Fractional amplitude of low-frequency fluctuations is disrupted in Alzheimer’s disease with depression. *Clin. Neurophysiol.* 128 1344–1349. 10.1016/j.clinph.2017.05.003 28570868

[B37] Zuo X.-N. Xu T. Jiang L. Yang Z. Cao X. Y. He Y. (2013). Toward reliable characterization of functional homogeneity in the human brain: preprocessing, scan duration, imaging resolution and computational space. *Neuroimage* 65 374–386. 10.1016/j.neuroimage.2012.10.017 23085497 PMC3609711

