# Corrigendum: Regional homogeneity in patients with mild cognitive impairment: A resting-state functional magnetic resonance imaging study

**DOI:** 10.3389/fnagi.2022.1098983

**Published:** 2022-12-13

**Authors:** Yu-Qian Wu, Yi-Ning Wang, Li-Juan Zhang, Li-Qi Liu, Yi-Cong Pan, Ting Su, Xu-Lin Liao, Hui-Ye Shu, Min Kang, Ping Ying, San-Hua Xu, Yi Shao

**Affiliations:** ^1^Department of Ophthalmology and Neurology, The First Affiliated Hospital of Nanchang University, Nanchang, China; ^2^Department of Ophthalmology, Massachusetts Eye and Ear, Harvard Medical School, Boston, MA, United States; ^3^Department of Ophthalmology and Visual Sciences, The Chinese University of Hong Kong, Sha Tin, Hong Kong SAR, China

**Keywords:** rs-fMRI, mild cognitive impairment, ReHo, spontaneous brain activity, Alzheimer's disease

In the published article, there was an error in Table 2 as published. The data of subject three was included in the previous data processing of demographics. Due to the excessive head movement of subject three in the fMRI data acquisition, the data of subject three needs to be deleted, so the following contents need to be corrected. The corrected Table 2 and its caption appear below.

**Table 2:** Demographic characteristic of the enrolled subjects.

**Condition**	**MCI**	**HC**	**t**	**P**
Subject	11	12	NA	NA
Age (y)	64.27 ± 7.35	64.00 ± 6.18	0.097	0.924
Gender (M:F)	5:6	6:6	NA	NA
Duration (month)	13.55 ± 9.59	0	4.687	< 0.001
SBP	128.45 ± 12.47	130.5 ± 10.83	−0.421	0.678
DBP	75.27 ± 12.39	76.17 ± 10.57	−0.187	0.854
HR	69.27 ± 9.48	70.42 ± 8.36	−0.308	0.761
Barthel Index	99.55 ± 1.51	100	−1.000	0.341
Best-corrected V A-left eye	0.30 ± 0.10	0.23 ± 0.06	2.181	0.041
Best-corrected V A-right eye	0.26 ± 0.12	0.21 ± 0.08	1.311	0.204
S100β	2.76 ± 1.09	0.18 ± 0.09	8.211	< 0.001
MMSE	22.09 ± 4.11	27.83 ± 2.52	−4.081	< 0.001

MCI, mild cognitive impairment; HC, Healthy control; NA, not applicable; SBP, systolic blood pressure; DBP, diastolic blood pressure; HR, heart rate.

In the published article, there was an error. The data of subject three was included in the previous data processing of demographics. Due to the excessive head movement of subject three in the fMRI data acquisition, the data of subject three needs to be deleted. A correction has been made to **Results**, “*Demographics*,” Paragraph 1:

“Subject 3's data have been deleted due to excessive head movement. There was no significant difference in mean age between the MCI and HC groups (64.27 ± 7.35 and 64.00 ± 6.18 yr, respectively; *P* = 0.924). There was no significant difference in the male to female ratio between the MCI and HC groups. The average MMSE scores of the MCI group was 22.09 ± 4.11 (*P* < 0.001). The average duration of the MCI was 13.55 ± 9.59 months (*P* < 0.001). The average S100β of MCI was 2.76 ± 1.09 (*P* < 0.001). A detailed summary of the data is presented in Table 2.”

In the published article, there was an error. There was a mistake in the description of the signals in the MCI group. A correction has been made to **Discussion**, Paragraph 6:

“A major function of the superior temporal gyrus is extracting meaningful linguistic features from speech inputs, and is strongly modulated by learning knowledge and perceived goals (Zhongwei et al., 2017). There is some evidence that the right STG functions in allocentric neglect deficits (Mao et al., 2021). The significantly higher signal in this region in the MCI group in the present study may be related to the altered perception of language in patients with MCI.”

The authors apologize for this error and state that this does not change the scientific conclusions of the article in any way. The original article has been updated.
